# IgG N-glycosylation contributes to different severities of insulin resistance: implications for 3P medical approaches

**DOI:** 10.1007/s13167-025-00410-x

**Published:** 2025-04-15

**Authors:** Xiaohong Chen, Lois Balmer, Kun Lin, Weijie Cao, Ziyu Huang, Xiang Chen, Manshu Song, Yongsong Chen

**Affiliations:** 1 Department of Endocrinology and Metabolism, The First Affiliated Hospital of Shantou University Medical College, Shantou, 515041 Guangdong China; 2 School of Medical and Health Sciences, Edith Cowan University, Joondalup, Perth, 6027 Australia; 3 Chemistry and Chemical Engineering Guangdong Laboratory, Shantou, 515041 Guangdong China; 4 Institute for Glycome Study, Shantou University Medical College, Shantou, 515041 Guangdong China; 5 Department of Laboratory Medicine, The First Affiliated Hospital of Shantou University Medical College, Shantou, 515041 Guangdong China; 6 Health Care Centre, The First Affiliated Hospital of Shantou University Medical College, Shantou, 515041 Guangdong China

**Keywords:** IgG N-glycosylation, Insulin resistance severity, Metabolic disorder, Inflammation, Protective biomarker, Immunometabolism, Primary prevention strategy, Predictive preventive personalized medicine (PPPM / 3PM)

## Abstract

**Background:**

Reliable biomarkers capturing immunometabolic processes in insulin resistance (IR) remain limited. IgG N-glycosylation modulates immune responses and reflects metabolic disorders, yet its role in IR remains unclear. This study investigated its potential for early detection, risk stratification, and targeted prevention within the framework of predictive, preventive, and personalised medicine (PPPM/3PM).

**Methods:**

A total of 313 participants were categorized into three groups based on the homeostatic model assessment for insulin resistance (HOMA-IR): insulin-sensitive (HOMA-IR < 2.69 without diabetes, n = 75), mild IR (HOMA-IR ≥ 2.69 without diabetes, n = 155), and severe IR (HOMA-IR ≥ 2.69 with type 2 diabetes, n = 83). Canonical correlation analysis was conducted to explore the overall relationship between IgG N-glycosylation and IR-related inflammation, indicated by tumour necrosis factor-α, interleukin- 6, C-reactive protein, and adiponectin. Mediation analysis was performed to evaluate the effect of IgG N-glycans on IR. Ordinal logistic regression was used to assess the association between IgG N-glycans and IR severity, with discriminative power evaluated using receiver operating characteristic curves.

**Results:**

Pro-inflammatory IgG N-glycoforms, characterized by reduced sialylation and galactosylation, along with increased bisecting N-acetylglucosamine, were observed as IR severity increased. IgG N-glycosylation significantly correlated with inflammatory markers in the insulin-sensitive (*r* = 0.599, *p* < 0.05), mild (*r* = 0.461, *p* < 0.05), and severe (*r* = 0.666, *p* < 0.01) IR groups. IgG N-glycosylation significantly influenced IR (*β* = 0.406) partially via modulation of inflammation. Increased glycoforms FA2[6]G1 (OR: 0.86, 95% CI: 0.78–0.96) and A2G2S2 (OR: 0.88, 95% CI: 0.82–0.94) were associated with a lower IR risk, with respective area under the curves (AUCs) of 0.752, 0.683, and 0.764 for the insulin sensitive, mild, and severe IR groups.

**Conclusions:**

IgG N-glycosylation contributes to IR by modulating inflammatory responses. Glycoforms FA2[6]G1 and A2G2S2 emerge as protective biomarkers, offering potential for predicting and preventing IR through primary prevention strategies within the PPPM framework.

**Supplementary Information:**

The online version contains supplementary material available at 10.1007/s13167-025-00410-x.

## Introduction

Insulin resistance (IR) is a key pathological feature of type 2 diabetes and metabolic syndrome, contributing to cardiovascular diseases and other metabolic complications [1, 2]. Despite advancements in diabetes management, early detection and prevention of IR remain challenging. Traditional markers, such as fasting glucose and insulin levels, often fail to capture disease onset and progression at early stages [3].

Chronic low-grade inflammation originating in adipose tissue is a primary driver of IR [4]. Adipose tissue dysfunction shifts the immune environment towards a pro-inflammatory state, increasing immune cell infiltration and cytokine secretion (e.g., tumour necrosis factor (TNF-α) and interleukin-6 (IL- 6)), which impair insulin signalling pathways [5, 6]. In contrast, adiponectin is an anti-inflammatory adipokine that enhances insulin sensitivity; however, its levels are reduced in obesity, further exacerbating IR [7, 8]. This inflammatory response extends beyond adipose tissue, leading to systemic inflammation characterized by elevated levels of C-reactive protein (CRP), which accelerates IR progression and increases type 2 diabetes risk [9]. These findings suggest that IR is not merely a metabolic disorder but also a chronic inflammatory condition involving immunometabolic dysregulation [5, 6], underscoring the need for novel biomarkers that capture the immune-metabolic interactions driving disease progression.

Predictive, preventive, and personalized medicine (PPPM/3PM) is an advanced healthcare paradigm focused on early disease prediction, targeted prevention, and personalized therapeutic strategies based on an individual’s unique biomolecular and clinical profile [10]. Given the multifactorial nature of IR, PPPM is increasingly recognized for its role in continuous health monitoring, early metabolic disturbance detection, and tailored prevention strategies [11–13]. Unlike conventional reactive treatments, PPPM stratifies high-risk individuals, addresses underlying disease drivers, and implements precision interventions to delay or prevent IR progression [14]. Integrating inflammatory and metabolic profiling facilitates patient stratification and targeted interventions, moving beyond generalized treatment approaches [15, 16]. Identifying biomarkers that reflect immune-metabolic alterations preceding overt metabolic dysfunction is crucial for improving early detection and risk stratification.

In this context, immunoglobulin G (IgG) N-glycosylation has emerged as a potential biomarker in metabolic diseases due to its role in modulating inflammatory response [17, 18, 22–24, 37, 38, 41, 77, 78]. Recent studies indicate that IgG accumulation in adipose tissue is linked to aging and metabolic decline, exacerbating adipose tissue inflammation and potentially contributing to IR [19, 20]. IgG mediates inflammatory response through its diverse N-glycan moieties, which vary across immune states and modulate the binding affinity of IgG fragment crystallizable (Fc) to Fc receptors [21]. Certain N-glycoforms of IgG drive pro-inflammatory states, perpetuating chronic inflammation in metabolic disorders, particularly as potential biomarkers for type 2 diabetes progression and related complications [18, 22, 24, 37, 46, 78, 38 ]. While prior studies have demonstrated IgG N-glycosylation is altered in metabolic disorders, most studies have only established a general association between glycan modifications and metabolic dysfunction [23, 24, 38, 41]. However, IR represents a distinct pathophysiological process within metabolic disorders, characterized by chronic low-grade inflammation and impaired insulin signalling [1]. The specific contribution of IgG N-glycans to IR progression remains poorly characterized.

Beyond metabolic diseases, IgG N-glycosylation is a recognized biomarker in autoimmune diseases and cancer, where glycan alterations influence immune activation, inflammation, and disease progression [25, 26, 79]. Given its immunomodulatory function [21], IgG N-glycosylation may contribute to IR by regulating inflammatory pathways. Identifying IgG N-glycan signatures associated with IR severity could provide insights into the inflammatory mechanisms driving disease progression. These glycan profiles could serve as novel biomarkers for early detection, prevention, and personalized interventions, aligning with PPPM principles.

In the context of PPPM, IgG N-glycans hold promise as early predictive biomarkers of IR, enabling risk stratification and targeted disease monitoring. Certain N-glycoforms enhance IgG Fc binding to immune cells, triggering the release of inflammatory markers such as TNF-α and IL- 6, thereby promoting chronic inflammation [36]. This inflammatory cascade is central to IR pathogenesis [5]. Since IgG N-glycosylation is a modifiable trait, its alterations could serve as therapeutic targets for early intervention and personalized therapeutic strategies, reinforcing its relevance to PPPM [12].

This case–control study investigates changes in IgG N-glycosylation profiles across varying severities of IR, categorized as insulin-sensitive, mild IR, and severe IR, and explores their interactions with key inflammatory markers associated with IR. Understanding these relationships may facilitate early disease prediction, inform preventive strategies, and pave the way for personalized interventions targeting IgG N-glycan-mediated inflammatory pathways.

## Methods

### Study design and participants

A total of 313 Han Chinese participants from the eastern Guangdong area of China were included in this case-control study, comprising 75 insulin-sensitive individuals, 155 with mild IR, and 83 with severe IR. IR was assessed using the HOMA-IR index, calculated as follows: HOMA-IR = fasting glucose (mmol/L) × fasting serum insulin (U/mL)/22.5 [28, 29]. Type 2 diabetes was diagnosed according to World Health Organization Criteria [81]. Participants without diabetes were defined as those who self-reported no history of diabetes and had a fasting glucose < 7.0 mmol/L and an HbA1c < 6.0%. Participants with HOMA-IR < 2.69 [30] and without diabetes were classified as insulin-sensitive, while those with HOMA-IR ≥ 2.69 and without diabetes were classified as having mild IR. Participants with HOMA-IR ≥ 2.69 and a diagnosis of type 2 diabetes were classified as having severe IR.

The included participants met the following criteria: (1) provided signed informed consent prior to participation and (2) aged over 20 years old. Individuals were excluded from the study based on the following criteria: (1) diagnosis of type 1 diabetes mellitus; (2) use of insulin; (3) presence of endocrine diseases such as hyper- or hypothyroidism or pituitary tumours; (4) being pregnant or breastfeeding; (5) diagnosis of malignant tumours or autoimmune diseases; (6) history of diabetic ketosis or ketoacidosis within 3 months prior to enrolment; (7) presence of acute conditions such as myocardial infarction, cerebrovascular accident, or serious infection within 1 month prior to enrolment; (8) planned or undergoing surgery during the study period; (9) use of medications such as glucocorticoids that interfere with glycaemic control; (10) history of psychiatric disorders or neurosis; and (11) inability to comply with the study protocol.

### Data collection and laboratory analyses

Demographic characteristics, including age and sex, were recorded during an initial interview. Phenotypic data, including height and weight, were measured to calculate body mass index (BMI). Participants fasted overnight for at least 8 h prior to blood sample collection. Fasting plasma glucose (FPG) levels were measured using the hexokinase method on the AU5800 chemistry analyser (Beckman Coulter, USA). Serum insulin concentrations were quantified using the chemiluminescence method on the ADVIA Centaur XP immunoassay analyser (Siemens, Germany). HbA1c levels were assessed via high-performance liquid chromatography on the HLC- 723G8 automated glycohemoglobin analyser (Tosoh, Japan). Total cholesterol, high-density lipoprotein (HDL) cholesterol, low-density lipoprotein (LDL) cholesterol, and triglycerides were measured on the AU5800 chemistry analyser (Beckman Coulter, USA) using the cholesterol oxidase method, direct method with hydrogen peroxide clearance, direct method with surfactant clearance, and glycerol phosphate oxidase method, respectively. Levels of TNF-α, IL- 6, CRP, and adiponectin were measured using the Human TNF-α ELISA kit (Catalog LV10514M, Animaluni, China), Human IL- 6 ELISA kit (Catalog LV10306M, Animaluni, China), Human CRP ELISA kit (Catalog LV10130M, Animaluni, China), and Human Adiponectin ELISA kit (Catalog LV10026M, Animaluni, China), respectively. All samples were randomized prior to testing, and laboratory personnel were blinded to participant status.

Protein G monolithic plates (BIA Separations), featuring a polymethacrylate monolithic stationary phase with immobilized G protein, were prepared for serum IgG isolation. Prior to isolation, protein G monolithic plate was washed with 2 mL ultra-pure water and equilibrated with 2 mL 1 × PBS (pH 7.4), followed by 1 mL of 0.1 M formic acid (pH 2.5). The plate was then re-equilibrated with 2 mL 10 × PBS once, and 2 mL 1 × PBS twice. Serum samples (100 μL) were diluted 1:7 (v/v) with 1 × PBS and filtered using an AcrPrep GHP 0.45 μm filter plate (PALL, Puerto Rico), then applied to the prepared protein G monolithic plate. Filtration of the samples was completed in 10 min and the plate was then washed three times with 1 × PBS to remove unbound proteins. IgG was released from the protein G monoliths using 1 mL of 0.1 M formic acid (pH 2.5). Eluates were collected in a 96-deep-well plate and immediately neutralized to pH 7.0 with 170μL of 1 M ammonium bicarbonate to preserve IgG stability. N-glycans were released from purified IgG samples and labelled using the 'in solution' method [31]. The isolated IgG samples were immobilized in a block containing 1.33% sodium dodecyl sulfate (SDS), 4% Igepal, and 5 × PBS, and N-glycans were released by digestion with recombinant N-glycosidase F (Roche, Germany) overnight at 37 °C. The released glycans were labelled with 2-aminobenzamide (Sigma) and purified with 96% acetonitrile (Sigma-Aldrich) and ultrapure water. Finally, IgG N-glycans were analysed by hydrophilic interaction liquid chromatography (HILIC) using an ultra-performance liquid chromatography (UPLC) system [31]. Glycoform quantification was performed to analyse 24 chromatographic glycan peaks of the IgG N-glycome. Glycan peak (GP) 3 was excluded from all the calculations because in some samples it co-eluted with a contaminant that significantly affected its value. Each GP was represented by alphanumeric characters indicating the absence or presence of galactose (G), core fucose (F), bisecting N-acetylglucosamine (GlcNAc) (B) or sialic acid (S) added to the core structure (Supplementary Table S1).

### Statistical analysis

The relative abundance of each directly measured glycoform was presented as a percentage of the total integrated peak area. Derived glycan traits were calculated from these directly measured glycans (Supplementary Table S1). All glycan data were *z*-standardized to ensure comparability and improve the performance of subsequent analyses. The normality of continuous data was assessed using the Shapiro–Wilk test. Continuous variables were described using the median and interquartile range and compared across the three groups using Kruskal–Wallis H test, followed by Mann–Whitney U tests for pairwise comparisons due to the skewed distribution. Categorical variables were expressed as frequencies and percentages and compared using Chi-squared tests. False discovery rate was controlled using Benjamini–Hochberg procedure.

Canonical correlation analysis (CCA) was performed to explore multivariate correlations between IgG N-glycoforms and IR phenotypes, represented by a panel of IR-related metabolic traits (FPG, fasting insulin, HbA1c, triglycerides, HDL cholesterol, LDL cholesterol, BMI), and inflammatory markers (TNF-α, IL- 6, CRP, adiponectin). Only IgG N-glycoforms that differed significantly among the three groups were included in the CCA. Subsequently, sparse multiset CCA (smCCA) was performed to identify the canonical variates for the three datasets: IgG N-glycoforms, metabolic traits, and inflammatory markers. Optimal tuning parameters were determined using a permutation test with 1,000 permutations, and the optimal penalties were selected based on the minimum *p*-value from the permutation results (R package “*PMA*”) [32]. The canonical weights obtained from smCCA were applied to derive the canonical variates [33], which were then used in mediation analysis (R package “*mediation*”). The mediation analysis tested whether IgG N-glycosylation influences IR-related metabolic traits by modulating inflammatory markers. Indirect, direct, and total effects of the mediation analysis were computed using a bootstrap procedure with 10,000 resamples, with significance defined as *p* < 0.05. The least absolute shrinkage and selection operator (LASSO) with 10-fold cross-validation was used (R package “*glmnet*”) to reduce data dimensionality and select relevant glycans. The glycans identified by LASSO were then included into stepwise regression alongside potential confounders, including age, sex, BMI, triglycerides, and HDL cholesterol [34]. Ordinal logistic regression models (R package “*MASS*”) were used to examine associations between IgG N-glycans and IR severity. Three ordinal logistic regression models were considered: (1) an unadjusted model, (2) a minimally adjusted model (age and sex), and (3) a fully adjusted model (age, sex, BMI, triglycerides, and HDL cholesterol). Triglycerides remained significant in the adjusted model. But given that both HDL cholesterol and BMI have been characterized as risk factors for IR and type 2 diabetes, they were retained in the fully adjusted model to account for potential confounding [34]. To evaluate the discriminatory ability of IgG N-glycoforms in distinguishing IR severity, receiver operating characteristic (ROC) curves were generated using the R package “*multiROC*”. The area under the curve (AUC) values were calculated to summarize the overall model performance. All statistical analyses and visualizations were conducted using R (version 4.3.1).

## Results

### Characteristics of study participants

Distinct physiological features were observed among the three groups with varying severities of IR (Table 1). Participants with severe IR showed significantly higher levels of BMI (*p* < 0.001), FPG (*p* < 0.001), HbA1c (*p* < 0.001), triglycerides (*p* < 0.001), LDL cholesterol (*p* < 0.05), CRP (*p* < 0.01) and adiponectin (*p* < 0.01), but lower levels of HDL cholesterol (*p* < 0.001), compared to those in the other two groups. Participants with mild IR had higher levels of fasting insulin (*p* < 0.01), TNF-α (*p* < 0.001), and IL- 6 (*p* < 0.001), but lower levels of CRP (*p* < 0.001) and adiponectin (*p* < 0.05), compared to the other groups. Overall, the levels of fasting insulin, HbA1c, LDL cholesterol, and inflammatory markers differed significantly, not only between insulin-sensitive participants and those with IR but also between participants with mild and severe IR. No significant differences were observed in age, sex, and total cholesterol among the three groups.

**Table 1:** Characteristics of the study participants

	Insulin-sensitive (*n* = 75)	Mild IR (*n* = 155)	Severe IR (*n* = 83)	p*
Sex (Male, n (%))	41 (54%)	84 (54.2%)	47 (56.6%)	0.936
Age (years)	52 (39–60)	50 (43–62)	52 (45–60)	0.605
BMI (kg/m^2^)	22.63 (20.75–24.14)	22.47 (20.94–23.20)	24.49 (22.94–26.70) ^a, b^	< 0.001
HOMA-IR	0.76 (0.20–1.29)	6.69 (5.07–9.03) ^a^	8.50 (5.15–13.81) ^a^	< 0.001
FPG (mmol/L)	5.43 (4.98–5.85)	5.49 (5.26–5.76)	9.15 (7.31–12.98) ^a, b^	< 0.001
HbA1c (%)	5.38 (5.11–5.55)	5.54 (5.31–5.72) ^a^	9.81 (7.49–12.34) ^a, b^	< 0.001
Fasting insulin (pmol/L)	2.84 (0.90–4.79)	27.11 (20.79–37.07) ^a^	23.30 (13.51–30.76) ^a, b^	< 0.001
Total cholesterol (mmol/L)	5.05 (4.37–5.80)	5.14 (4.70–5.82)	5.38 (4.72–5.90) ^a^	0.192
Triglyceride (mmol/L)	1.06 (0.80–1.52)	1.02 (0.77–1.40)	2.08 (1.29–3.12) ^a, b^	< 0.001
HDL cholesterol (mmol/L)	1.36 (1.08–1.59)	1.38 (1.17–1.67)	1.10 (0.94–1.38) ^a, b^	< 0.001
LDL cholesterol (mmol/L)	2.77 (2.39–3.52)	3.15 (2.61–3.66) ^a^	3.37 (2.81–3.76) ^a, b^	< 0.01
CRP (mg/L)	0.76 (0.24–1.86)	0.02 (0.02–0.03)^a^	3.00 (1.52–4.80)^a, b^	< 0.001
TNF-α (pg/mL)	30.77 (18.34–56.63)	183.94 (155.61–226.94) ^a^	148.63 (35.96–189.93) ^a, b^	< 0.001
IL-6 (pg/mL)	24.41 (19.24–34.20)	35.54 (25.47–44.26) ^a^	23.70 (17.57–28.50) ^b^	< 0.001
Adiponectin (ng/mL)	0.70 (0.55–0.89)	0.67 (0.50–0.83) ^a^	0.80 (0.70–0.94) ^a, b^	< 0.001

*IR* insulin resistance, *BMI* body mass index, *HOMA-IR* homeostatic model assessment for insulin resistance, *FPG* fasting plasma glucose, *HbA1c* haemoglobin A1c, *HDL* high-density lipoprotein, *LDL* low-density lipoprotein, *CRP* C-reactive protein, *TNF-α* tumour necrosis factor-α, *IL-6* interleukin-6. *The *p* value represents the comparison among the three groups. ^a^Statistically significant compared with the insulin-sensitive group. ^b^Statistically significant compared with the mild IR group

### Changes in IgG N-glycosylation profiles across IR severities

**Fig. 1:**
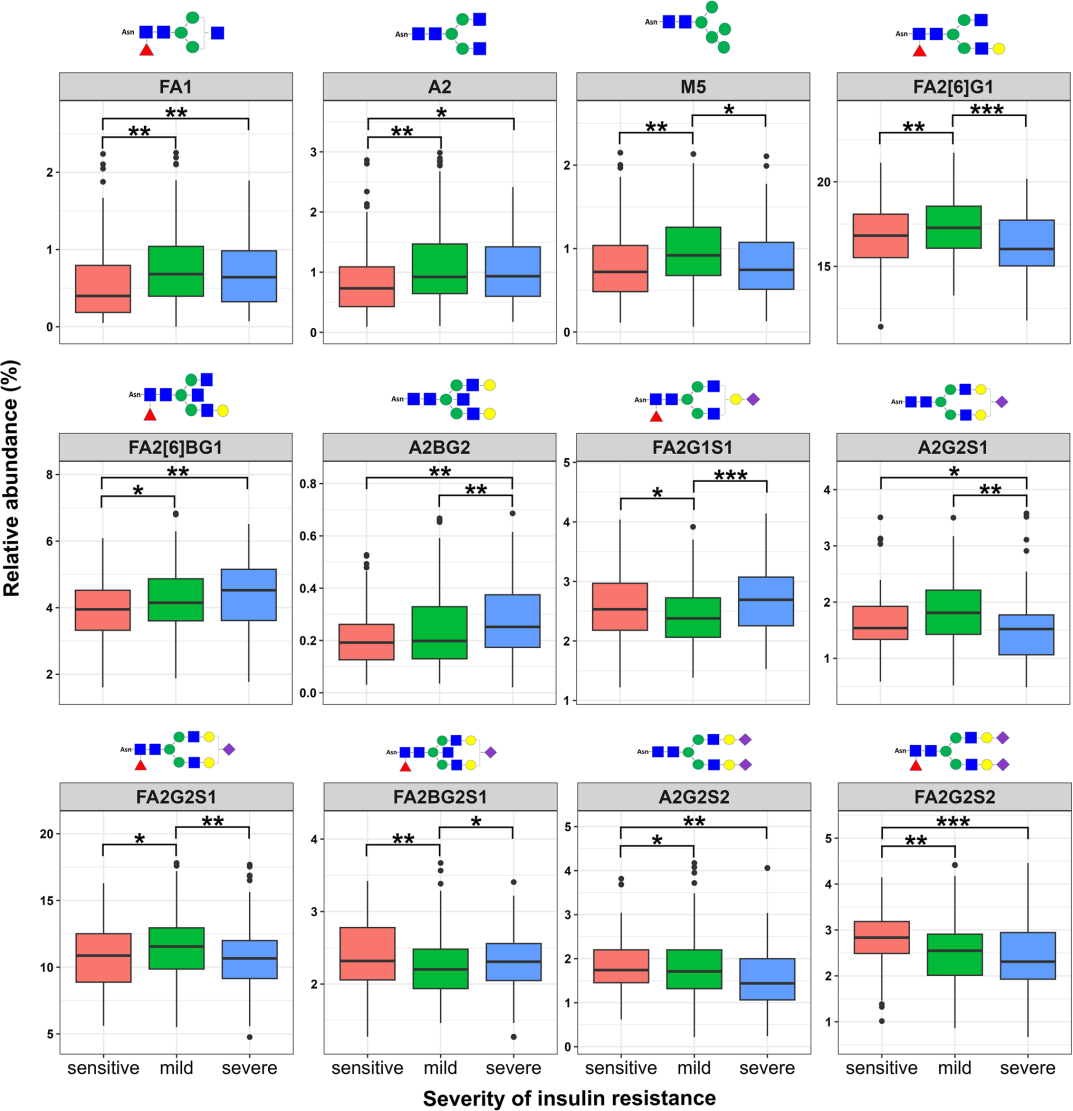
Significant alterations in twelve IgG N-glycoforms across different severities of insulin resistance (IR). Each panel represents a specific IgG N-glycoform with significant differences across the three groups (p < 0.05). The schematic structures of the glycoforms are displayed on the top of each panel. The x-axis indicates the groups: insulin-sensitive, mild IR, and severe IR. The y-axis indicates the abundance of each glycoform. F, α−1, 6-linked core fucose; A_x_, number (x) of antennae; B, bisecting N-acetylglucosamine (GlcNAc) β (1–4) linked to β (1–3) mannose; M_x_, number (x) of mannose residues; G_x_, number (x) of β (1–4)-linked galactoses; [6]G1, galactose on the antenna of the α(1–6)-linked mannose; S_x_, number (x) of sialic acids linked to galactose. The structural schemes of IgG N-glycans are visually represented using symbols: GlcNAc (blue square), mannose (green circle), fucose (red triangle), galactose (yellow circle), and sialic acid (purple rhomb). Statistical significance is indicated as * *p* < 0.05, ** *p* < 0.01, *** *p* < 0.001

Significant differences in IgG N-glycosylation profiles were observed among the three groups with varying IR severities (Fig. 1, Supplementary Table S2-S3). Both the mild and severe IR groups demonstrated a decline in disialylation (S2) (*p* < 0.001) and an increase in asialylation (*p* < 0.05) compared to the insulin-sensitive group. Agalactosylation levels were higher in severe IR group (*p* < 0.05), whereas digalactosylation (G2) levels were lower (*p* < 0.05), compared to the insulin-sensitive group. An escalating trend in bisecting GlcNAc (B) was observed as the severity of IR increased (*p* < 0.05) (Supplementary Table S3).

Complex-type biantennary N-glycan structures (A2) exhibited predominant alterations in IgG N-glycosylation profiles across the three groups. In the severe IR group, asialylated digalactosylated glycans with bisecting GlcNAc (A2BG2) exhibited the highest levels (*p* < 0.01), while monosialylated digalactosylated glycans without bisecting GlcNAc (A2G2S1) showed the lowest levels (*p* < 0.05) (Fig. 1, Supplementary Table S2). In contrast, in the mild IR group, monosialylated digalactosylated glycans with core fucosylation (FA2G2S1) increased (*p* < 0.05), whereas monosialylated digalactosylated glycans with core fucosylation and bisecting GlcNAc (FA2BG2S1) decreased (*p* < 0.05) (Fig. 1, Supplementary Table S2). Additionally, galactosylated glycans without sialylation or bisecting GlcNAc, such as FA2[6]G1 (*p* < 0.01) and mannose- 5 (M5) glycans (*p* < 0.05), exhibited the highest levels in the mild IR group, whereas monogalactosylated glycans with monosialylation (FA2G1S1) were at their lowest levels (*p* < 0.05) (Fig. 1, Supplementary Table S2). Compared to both mild and severe IR groups, the insulin-sensitive group showed the highest levels of digalactosylated disialylated glycans, including FA2G2S2 (*p* < 0.01) and A2G2S2 (*p* < 0.05). In contrast, it exhibited the lowest levels of agalactosylated asialylated glycans (e.g., FA1, A2, FA2B, *p* < 0.05) and monogalactosylated glycans with core fucosylation and bisecting GlcNAc (e.g., FA2[6]BG1, FA2[3]BG1, *p* < 0.05) (Supplementary Table S2). Both FA2[6]G1 and A2G2S2 showed a trend of higher levels in the insulin-sensitive and mild IR groups compared to the severe IR group, though the increase in FA2[6]G1 in the insulin-sensitive group and A2G2S2 in the mild IR group were not statistically significant (Fig. 1).

### IgG N- glycosylation is correlated with immunometabolism in IR

**Fig. 2:**
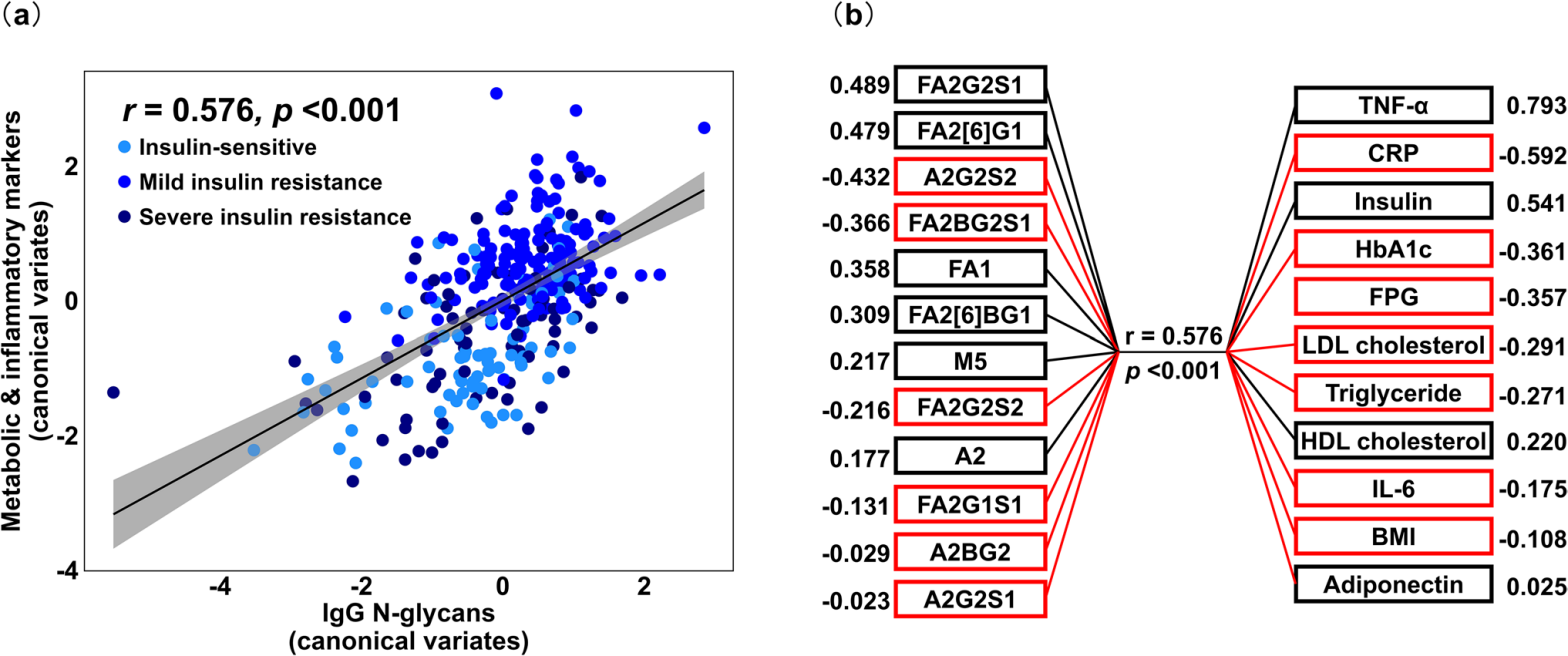
Canonical correlations between IgG N-glycoforms and immunometabolic traits in insulin resistance (IR). **a** The scatter plot illustrates the first pair of canonical correlations, showing a positive canonical correlation (*r* = 0.576,* p* < 0.001) between IgG N-glycans and IR-related metabolic and inflammatory markers. The x- and y-axes represent the canonical variates for each of the two datasets, respectively. The black line represents the line of best fit, indicating positive correlations. Individual data points are categorized by three severity levels of IR, with corresponding colours. **b** The schematic plot presents the canonical loadings of each variable in the canonical correlations. The numerical values for each variable indicate the canonical loadings, representing each variable’s contribution to the canonical correlation. Higher absolute values suggest a greater contribution to the relationship between the two sets of variables. All variables are sorted by the absolute values of their canonical loadings. Positive relationships are shown in black, while negative relationships are in red. F, α−1, 6-linked core fucose; A_x_, number (x) of antennae; B, bisecting N-acetylglucosamine (GlcNAc) β (1–4) linked to β (1–3) mannose; M_x_, number (x) of mannose residues; G_x_, number (x) of β (1–4)-linked galactoses; [6]G1, galactose on the antenna of the α (1–6)-linked mannose; S_x_, number (x) of sialic acids linked to galactose; BMI, body mass index; HOMA-IR, homeostatic model assessment for IR; FPG, fasting plasma glucose; HbA1c, haemoglobin A1c; HDL, high-density lipoprotein; LDL, low-density lipoprotein; CRP, C-reactive protein; TNF-α, tumour necrosis factor-α; IL- 6, interleukin- 6

To further elucidate the role of IgG N-glycosylation in the immunometabolic regulation of IR, CCA was applied to examine the overall relationships between IgG N-glycoforms and a panel of metabolic and inflammatory markers related to IR (Fig. 2). The first two canonical variates were significantly correlated, with canonical correlations of 0.576 (*F* = 2.15, *p* < 0.001) and 0.404 (*F* = 1.42, *p* < 0.01), respectively (Fig. 2a, Supplementary Tables S4 - 5). Variables with canonical loadings >| 0.3| were considered significant contributors to the canonical correlations (Fig. 2b). Six IgG N-glycoforms exhibited significant loadings: FA2G2S1 (0.489), FA2[6]G1 (0.479), A2G2S2 (− 0.432), FA2BG2S1 (− 0.366), FA1 (0.358), and FA2[6]BG1 (0.309). Fucosylated glycans with reduced galactosylation or sialylation (e.g., FA2G2S1, FA2[6]G1, FA1, and FA2[6]BG1) demonstrated positive loadings to the canonical correlations, while digalactosylated disialylated glycans (A2G2S2) and monosialylated digalactosylated glycans with core fucosylation and bisecting GlcNAc (FA2BG2S1) showed negative loadings. Among the immunometabolic traits, TNF-α (0.793), CRP (− 0.592), insulin (0.541), HbA1c (− 0.361), and FPG (− 0.357) were significantly correlated with IgG N-glycoforms. These results demonstrate that IgG N-glycosylation profiles are significantly correlated with IR related immunometabolic traits.

### Contribution of IgG N-glycosylation to inflammatory states in IR

**Fig. 3:**
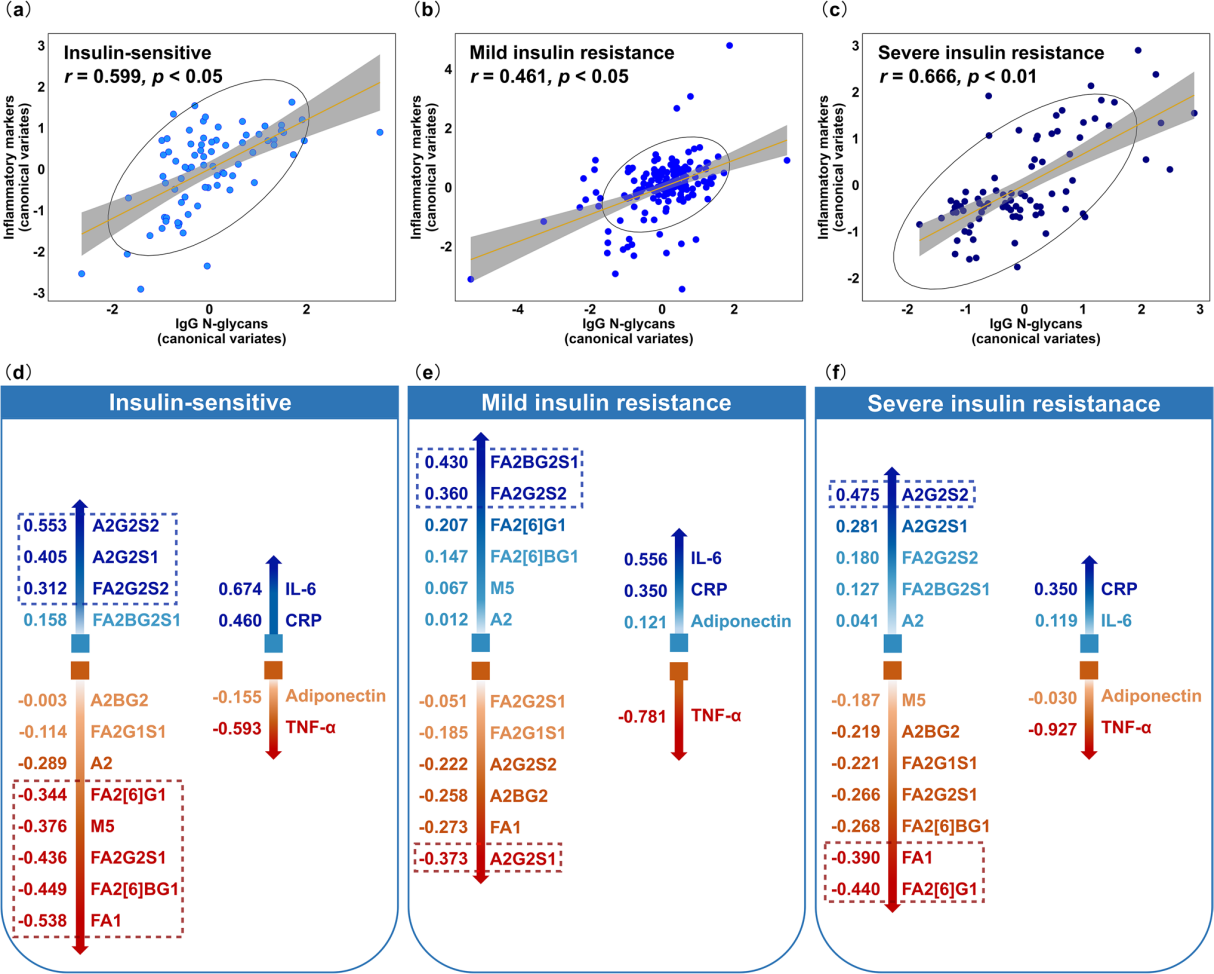
Canonical correlations between IgG N-glycans and inflammatory markers across the three severity levels of insulin resistance (IR). **a**-**c** The scatter plots illustrate the first pairs of canonical correlations, showing positive canonical correlations between IgG N-glycans and IR-related inflammatory markers for (**a**) the insulin-sensitive group (*r* = 0.599, *p* < 0.05), (**b**) the mild IR group (*r* = 0.461, *p* < 0.05), and (**c**) the severe IR group (*r* = 0.666, *p* < 0.01). The x- and y-axes represent canonical variates for each of the two datasets, respectively. The black line represents the line of best fit, indicating positive correlations. **d-f** The schematic plots display canonical loadings of each variable in the canonical correlations between IgG N-glycans and inflammatory markers across the three levels of IR severity. The numerical values of each variable indicate the canonical loadings, representing each variable’s contribution to the canonical correlations. Higher absolute values suggest a greater contribution to the relationship between the two sets of variables. All variables are sorted by the absolute values of their canonical loadings, with corresponding colours. Positive relationships are shown in blue, while negative relationships are in red. F, α−1, 6-linked core fucose; A_x_, number (x) of antennae; B, bisecting N-acetylglucosamine (GlcNAc) β (1–4) linked to β (1–3) mannose; M_x_, number (x) of mannose residues; G_x_, number (x) of β (1–4)-linked galactoses; [6]G1, galactose on the antenna of the α (1–6)-linked mannose; S_x_, number (x) of sialic acids linked to galactose

To examine how the IgG N-glycosylation contributes to the inflammatory states associated with IR, canonical correlations between IgG N-glycans and inflammatory markers were analysed across varying IR severities. Significant canonical correlations were identified in the insulin-sensitive group (*r* = 0.599, *p* < 0.05, Fig. 3a, Supplementary Tables S6 - 7), the mild IR group (*r* = 0.461, *p* < 0.05, Fig. 3b, Supplementary Tables S8 - 9), and the severe IR groups (*r* = 0.666, *p* < 0.01, Fig. 3c, Supplementary Tables S10 - 11). In the insulin-sensitive group, the IgG N-glycoforms with the strongest loadings were A2G2S2 (0.553), FA1 (− 0.538), FA2[6]BG1 (− 0.449), FA2G2S1 (− 0.436), A2G2S1 (0.405), M5 (− 0.376), FA2[6]G1 (− 0.344), and FA2G2S2 (0.312) (Fig. 3d). In the mild IR group, the key IgG N-glycoforms were FA2BG2S1 (0.430), A2G2S1 (− 0.373), and FA2G2S2 (0.360) (Fig. 3e). In the severe IR group, the most influential glycoforms were A2G2S2 (0.475), FA2[6]G1 (− 0.440), and FA1 (− 0.390) (Fig. 3f).

The number of IgG N-glycoforms with significant loadings decreased noticeably in the mild and severe IR groups. Several glycoforms consistently displayed the same loading direction across all dimensions (e.g., FA2G2S1, FA2G2S2, FA2G1S1, FA1, and A2BG2), while others shifted the directions depending on the IR severity. Specifically, glycoforms M5, FA2[6]BG1, and FA2[6]G1 shifted from negative to positive in mild IR dimension, whereas A2G2S1 and A2G2S2 shifted from positive to negative. Additionally, the loadings of TNF-α increased from − 0.593 to − 0.781 and − 0.927 as the severity of IR intensified, demonstrating the strongest and most stable associations with the IgG N-glycans. In contrast, the loadings of IL- 6, CRP and adiponectin showed a decreasing trend (Fig. 3d-f).

### Independent influence of IgG N-glycans on IR: Partial mediation by inflammatory markers

**Fig. 4:**
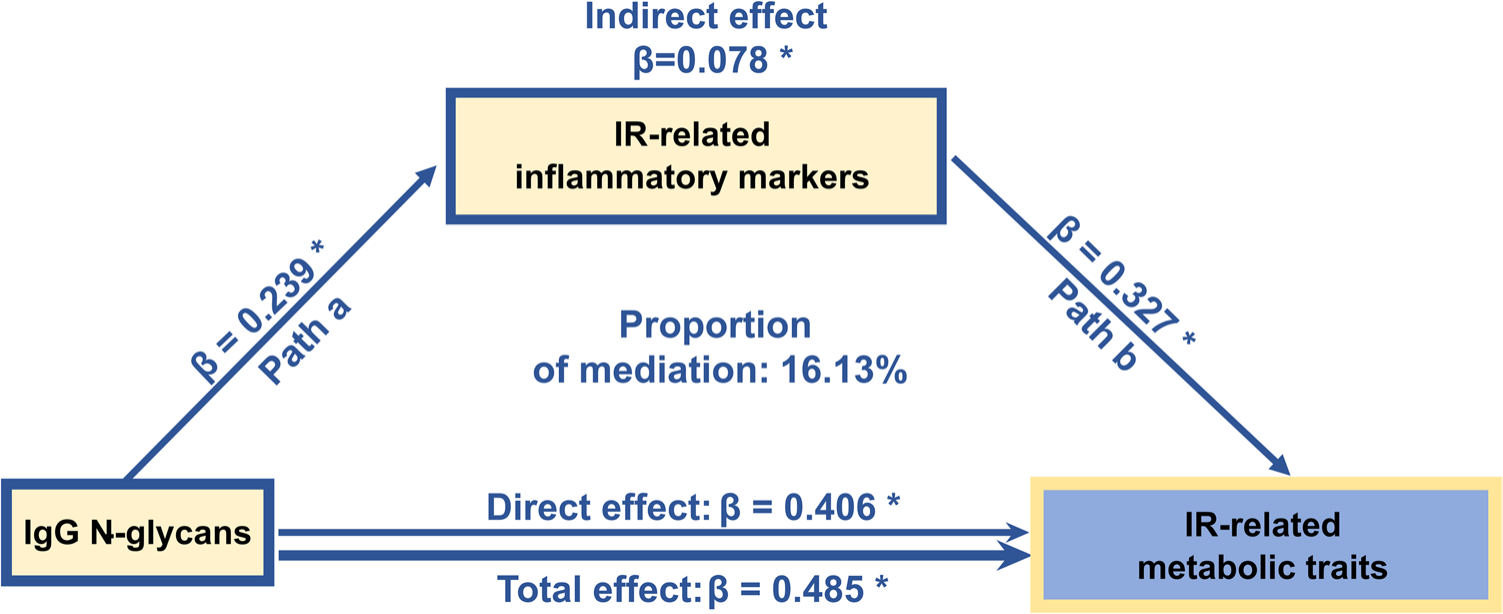
Independent influence of the IgG N-glycans on insulin resistance (IR). This figure illustrates the effect of the IgG N-glycans on IR-related metabolism, with partial mediation by inflammatory markers. The direct effect reflects the influence of IgG N-glycans on IR-related metabolism that is not mediated by inflammatory markers. The total effect represents the overall impact of IgG N-glycans on IR-related metabolism, incorporating all pathways. **Path a** represents the influence of IgG N-glycans on inflammatory markers, while **Path b** shows the effect of inflammatory markers on IR-related metabolism. All effects are represented by β values. Proportions of mediation are also shown, indicating the contributions of indirect effects to the total effects. * *p* < 0.001

Using canonical variates obtained from smCCA (Supplementary Tables S12 - 13), mediation analysis revealed that IgG N-glycans influenced IR-related metabolism in two ways: direct and indirect pathways, the latter mediated by inflammatory markers. IgG N-glycans exhibited significant direct, indirect, and total effects on IR-related metabolism (Fig. 4, Supplementary Table S14). Specifically, changes in IgG N-glycosylation independently influenced metabolic traits (direct effect: *β* = 0.406, *p* < 0.001). Additionally, IgG N-glycosylation affected IR indirectly (*β* = 0.078, *p* < 0.001) via its impact on inflammatory markers (Path a: *β* = 0.239, *p* < 0.001), which subsequently influenced metabolic traits (Path b: *β* = 0.327*, p* < 0.001). This indirect pathway accounted for 16.13% of the total effect of IgG N-glycans on IR.

### Distinct IgG N-glycoforms associated with IR severities

**Fig. 5:**
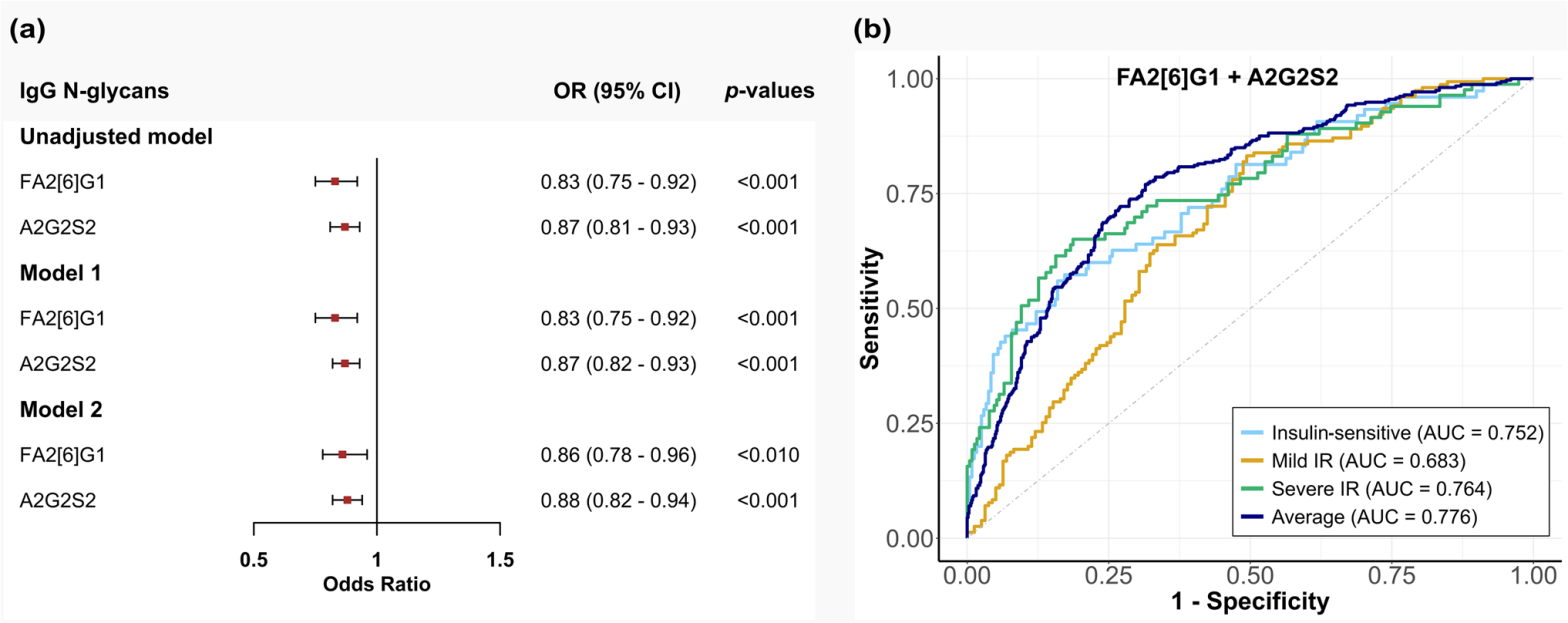
Summary of ordinal logistic regression model. The forest plot (**a**) represents the odds ratios (OR) of FA2[6]G1 and A2G2S2 for the insulin resistance (IR) severity across three ordinal logistic regression models: an unadjusted model, Model 1 (adjusted model for age and sex), and Model 2 (adjusted model for age, sex, BMI, triglycerides, and HDL cholesterol). The red points represent the estimated OR, and the horizontal lines through the red points represent the 95% confidence intervals (95% CI) for the OR. The *p*-values associated with each OR assess the statistical significance of each estimate. The ROC curve (**b**) shows the discriminative power of the fully adjusted model. The curve plots the true positive rate (sensitivity) against the false positive rate (1-specificity) for the three groups representing varying severities of IR. The area under the curve (AUC) values corresponding to each curve quantify the model’s ability to discriminate between IR severities. F, α−1, 6-linked core fucose; A_x_, number (x) of antennae; G_x_, number (x) of β (1–4)-linked galactoses; [6]G1, galactose on the antenna of the α (1–6)-linked mannose; S_x_, number (x) of sialic acids linked to galactose

To further confirm the association between IgG N-glycans and IR severity, an ordinal logistic regression model was developed using LASSO. Among the twelve IgG N-glycoforms that significantly altered across the three groups, seven glycoforms: FA1, FA2[6]G1, FA2[6]BG1, FA2G1S1, A2G2S1, A2G2S2, and FA2G2S2 were selected by LASSO (Supplementary Table S15). Higher levels of glycoforms FA2[6]G1 (OR: 0.86, 95% CI: 0.78–0.96, *p* < 0.01) and A2G2S2 (OR: 0.88, 95% CI: 0.82–0.94, *p* < 0.001) showed significant associations with a lower risk of IR, after adjusting for age, sex, BMI, triglycerides, and HDL cholesterol (Fig. 5a, Supplementary Table S16). These findings support the previous observation that FA2[6]G1 and A2G2S2 levels were higher in the insulin-sensitive and mild IR groups compared to the severe IR group, suggesting their potential protective role against IR progression. The AUC values for FA2[6]G1 and A2G2S2 were 0.752 (95% CI: 0.686–0.818) for insulin sensitive group, 0.683 (95% CI: 0.624–0.742) for mild IR group, and 0.764 (95% CI: 0.700–0.828) for severe IR group (Fig. 5b). With an average AUC value of 0.776, these glycoforms demonstrated moderate to good predictive power in distinguishing insulin-sensitive individuals from those with varying IR severities.

## Discussion

In this study, significant alterations in IgG N-glycosylation profiles were associated with the severity of IR. Specifically, pro-inflammatory IgG N-glycoforms with reduced sialylation, reduced galactosylation, and increased bisecting GlcNAc, were more prevalent as IR severity increased. These altered IgG N-glycans demonstrated significant canonical correlations with inflammatory markers, including TNF-α, IL- 6, CRP, and adiponectin, which could have mediated the influence of IgG N-glycans on IR. Additionally, higher levels of glycoforms A2G2S2 and FA2[6]G1 were associated with a lower risk of progressing to more severe IR. These findings provide critical insights into how specific IgG N-glycans contribute to the progression of IR and its associated inflammatory processes.

### Unmet needs in IR diagnosis and management

IR is a metabolic dysfunction commonly characterized by a pro-inflammatory state, where enhanced inflammation plays a pivotal role in its progression [35]. However, current diagnostic approaches primarily rely on clinical markers such as fasting glucose and insulin, which may not capture the underlying immunometabolic dysfunction at earlier stages [3]. Identifying novel biomarkers that reflect both metabolic and immune dysregulation could facilitate earlier detection and more precise risk stratification of IR. Recent findings found that IgG exacerbates adipose tissue inflammation, a primary driver of IR, suggesting its role in initiating IR [4, 19, 20]. Changes in IgG N-glycans can switch between pro-inflammatory and anti-inflammatory responses, influencing immune and metabolic processes [36]. Notably, several studies have documented associations between IgG N-glycosylation and metabolic diseases [18, 22–24, 37, 38, 41, 77, 78], yet its specific role in IR remains unclear.

### IgG N-glycosylation as a novel biomarker

IgG N-glycans interact with Fcγ receptors (FcγRs) on immune cells such as macrophages, neutrophils, and NK cells, triggering pro-inflammatory responses, like antibody-dependent cellular cytotoxicity (ADCC), complement-dependent cytotoxicity (CDC), and phagocytosis [36]. Upon recognition by complement component 1q (C1q), IgG-antigen complexes can initiate the classical complement pathway, forming the membrane attack complex (MAC) and triggering CDC [39]. Altered IgG N-glycosylation increases FcγRIIIA interactions on NK cells, promoting cytotoxic granule release and ADCC [40]. Lower levels of sialylation and galactosylation, along with higher bisecting GlcNAc in IgG N-glycoforms, have been associated with proinflammatory diseases, including type 2 diabetes, dyslipidaemia, and metabolic syndrome [18, 22–24, 37, 38, 77, 78]. While prior studies have reported similar trends in IgG N-glycosylation in type 2 diabetes, this study provides more detailed stratification by IR severity, including patients with type 2 diabetes and severe IR, and non-diabetic individuals with mild IR. Our findings reveal a stepwise shift in glycosylation patterns from the insulin-sensitive state to severe IR, characterized by increased bisecting GlcNAc and decreased sialylation and galactosylation. These findings align with evidence suggesting that the absence of galactose or sialic acid, or the presence of bisecting GlcNAc, enhances IgG affinity for FcγRs on immune cells, promoting a pro-inflammatory response [26, 42]. Consequently, asialylated IgG, agalactosylated IgG, and IgG with bisecting GlcNAc are potent mediators of pro-inflammation responses in IR [43].

Notably, the severe IR group exhibited higher levels of digalactosylated, asialylated glycans with bisecting GlcNAc, whereas the mild resistance group showed a predominance of galactosylated glycans lacking sialylation or bisection. This pattern suggests that IgG glycosylation may undergo further modifications as IR progresses, contributing to worsening metabolic dysfunction and inflammation [23]. The shift towards pro-inflammatory IgG N-glycoforms as IR severity increased highlights their integral roles in the pathogenesis and progression of IR [27]. By expanding upon previous studies that primarily examined type 2 diabetes cohorts, our study highlights how glycosylation patterns dynamically evolve across IR severity stages, reinforcing the potential of IgG N-glycosylation as a biomarker for disease progression [18, 22, 41].

### Potential for predictive and preventive strategies in the framework of PPPM

Importantly, the observed changes in the mild IR group suggest that IgG N-glycan alterations could serve as early indicators of IR, offering the potential for predictive biomarkers in the framework of PPPM [18]. Moreover, higher levels of glycans FA2[6]G1 and A2G2S2 were associated with a lower risk of progressing to more severe IR, indicating their potential as protective biomarkers. FA2[6]G1 has been linked to a lower risk of type 2 diabetes [44, 45]. In this study, FA2[6]G1 levels peaked in the mild IR group without type 2 diabetes and then declined in the severe IR group diagnosed with type 2 diabetes. This suggests that FA2[6]G1 may mark a transitional phase in disease development, reflecting shifts in IgG N-glycosylation as IR worsens. A2G2S2, characterized by high sialylation and galactosylation, possesses anti-inflammatory properties [36] and showed a steady decline with increasing IR severity in the current study. Its consistent association with lower IR severity suggests a potential protective role in mitigating the pro-inflammatory response during IR progression. Both FA2[6]G1 and A2G2S2 demonstrated strong performance in classification models, with an average AUC of 0.776, indicating moderate to good predictive power. These glycoforms effectively distinguish insulin-sensitive individuals from those with varying severities of IR, suggesting that FA2[6]G1 and A2G2S2 could serve as promising biomarkers for early-stage risk assessment and support an integrated approach to predicting and mitigating IR risk by enabling timely intervention at the earliest signs of disease progression [64, 65].

### IgG N-glycosylation as a modulator of immunometabolic regulation in IR

IgG N-glycosylation plays a crucial role in the interplay between inflammation and metabolic disorders [18, 23, 45], as corroborated by canonical correlations identified in the current study, reinforcing its involvement in the immunometabolic regulation of IR. In the insulin-sensitive group, moderate and significant correlations (*r* = 0.599) with multiple glycan structures suggest that IgG N-glycans play a role in maintaining inflammatory homeostasis, thereby preventing metabolic dysfunction [23, 46]. However, this balance appears to be disrupted in mild IR [46], as evidenced by a substantial reduction in IgG N-glycans with significant loadings, along with higher TNF-α and IL- 6 levels but lower CRP and adiponectin levels. At this stage, systemic inflammation may not be fully established, as indicated by lower CRP levels [47, 48]. IgG glycosylation may be shifting toward a pro-inflammatory profile in response to localized inflammation [47, 49], which leads to a still significant but weaker correlation (*r* = 0.461). The reduced adiponectin in mild IR further indicates early adipose tissue dysfunction, reinforcing a pro-inflammatory state that precedes type 2 diabetes [34]. As IR progresses, the correlation strengthened (*r* = 0.666), reflecting an increasing immune-metabolic interplay [47]. In the severe IR group, higher adiponectin and lower TNF-α and IL- 6 levels were observed despite a higher BMI, which may be influenced by anti-diabetic treatments. Data on anti-diabetic treatments were not collected in this study, so their potential impact on inflammatory markers and metabolic outcomes could not be assessed. However, these treatments may not fully counteract the pro-inflammatory response, as TNF-α and CRP levels remained elevated compared to the insulin-sensitive group.

Notably, significant correlations driven by fewer glycans emerged at the mild IR stage, marking an early transition in disease progression, where IgG glycosylation may have begun to amplify inflammation, ultimately driving the progression toward severe IR [18]. Mediation analysis in this study further supports the role of IgG N-glycans in modulating IR through inflammatory markers. Pro-inflammatory glycoforms of IgG can enhance the secretion of TNF-α and IL- 6 through the activation of FcγRs on immune cells [50, 51]. These elevated cytokines stimulate hepatic CRP synthesis [52, 53], which in turn amplifies inflammatory signalling and macrophage activation [53]. Furthermore, TNF-α, IL- 6 and CRP supress adiponectin gene expression and production [54, 55], preventing its anti-inflammatory effects and further worsening IR [55]. Collectively, these findings highlight IgG N-glycan alterations as both biomarkers and modulators of IR, aligning with PPPM principles.

### Clinical implications and future directions

The findings of this study have significant implications for PPPM, emphasizing the shift from reactive treatment to proactive health management [10]. By identifying specific IgG N-glycosylation profiles associated with varying severities of IR, this research contributes to the development of predictive biomarkers for early detection and risk stratification of IR, informing targeted preventive strategies within the PPPM framework [15]. Beyond their potential as biomarkers, IgG N-glycans may also serve as therapeutic targets. Studies suggest that manipulating IgG glycosylation can be therapeutic strategy to modulate inflammatory response for clinical applications [56, 57]. Strategies such as enhancing IgG sialylation through genetic, metabolic or chemoenzymatic modifications have shown promise in both preclinical and clinical studies for autoimmune and inflammatory conditions [57–59]. Immunosuppressive therapies have also been reported to restore IgG sialylation and galactosylation, attenuating inflammation in autoimmune diseases [42]. Furthermore, intravenous immunoglobulin (IVIG) therapy, which relies on IgG glycosylation, is already used in autoimmune and inflammatory conditions, highlighting the therapeutic relevance of glycan modulations [26, 60].

These insights open possibilities for pharmacological or glycoengineering approaches targeting IgG N-glycosylation to mitigate inflammation and IR progression [42]. The ability to modulate IgG glycosylation profiles offers a unique opportunity to manage both inflammation and metabolic dysfunction simultaneously [23]. Personalized medicine could leverage glycosylation profiling to tailor interventions based on individual glycan and inflammatory marker profiles, improving IR management [61]. Integrating IgG N-glycosylation analysis into clinical practice aligns with the PPPM framework [11, 18, 37], allowing for a more individualized approach, enabling individualized monitoring, early therapeutic interventions, and more precise management of IR. For example, personalized treatments could involve monitoring IgG N-glycosylation changes over time and adjusting therapeutic strategies to prevent progression to type 2 diabetes [18].

However, translating glycosylation-based therapies into clinical practice faces challenges. These include the complexities of targeting specific glycan modifications, potential variability in responses to glycoengineering interventions, and the need for robust, standardized methods for glycan profiling [26, 42, 62]. Moreover, under the newly proposed “paracentral dogma” theory, glycans are recognized as the 3^rd^ life code after DNA/RNA (the 1^st^) and amino acids (the 2^nd^), interacting with genetic predisposition, environmental factors (e.g., diet, physical activity), and comorbid conditions (e.g., obesity, chronic infections, autoimmune diseases) [17, 80]. These interactions must be considered, as they influence IgG N-glycosylation profiles and pose additional challenges in standardizing glycan-based biomarkers for clinical applications [26]. While inter-individual variability complicates biomarker standardization [26], understanding these factors may uncover opportunities for more tailored therapeutic approaches [61]. By integrating glycosylation profiling into the broader framework of PPPM, which emphasizes preventive care and personalized treatment, healthcare systems can move toward more precise, effective management strategies for IR and metabolic diseases [11, 13, 14].

## Limitations

This study did not capture the longitudinal progression of IR, limiting causal inferences despite categorizing participants by severity. Validation in larger, more diverse cohorts is needed to confirm generalizability beyond Han Chinese population and to include participants from various ethnic backgrounds, ensuring broader applicability of the findings. Future longitudinal studies should assess how dynamic changes in IgG N-glycosylation influence inflammation and IR over time. Additionally, data on oral anti-diabetic medication use, diet, and physical activity were not collected, which may have influenced inflammatory markers and metabolic outcomes [26]. Future studies should take these factors into account to better interpret group differences. Additionally, incorporating glycomics with other advanced omics approaches, such as proteomics and metabolomics, into the PPPM framework could enhance biomarker discovery and monitoring [61]. These integrative strategies could refine PPPM-based management of IR, leading to more precise and proactive interventions.

## Conclusion and expert recommendations

This study is the first to demonstrate significant alterations in IgG N-glycosylation profiles across varying severity levels of IR and their associations with inflammatory processes. Pro-inflammatory IgG N-glycans were linked to increased IR severity, likely through their modulation of inflammatory markers. Notably, higher levels of FA2[6]G1 and A2G2S2 were associated with lower IR severity, highlighting their potential as predictive markers for risk assessment and preventive strategies. Within the framework of PPPM, leveraging these biomarkers could enhance early detection, facilitate risk stratification, and support primary prevention efforts for IR.

## PPPM innovation highlights

### Working hypothesis in the framework of PPPM

Chronic inflammation is a key driver of IR, yet its mechanistic underpinnings remain unclear [63]. The study hypothesized that specific IgG N-glycans modulate inflammatory responses implicated in the development of IR, with glycosylation changes associated with varying severities of IR. These alterations contribute to the immunometabolism regulation, influencing the interaction between inflammatory responses and metabolic dysfunction. By integrating immunometabolic biomarkers, including IgG N-glycans, inflammatory cytokines, and metabolic traits, into the PPPM framework, this study provides new insights into the pathophysiology of IR. This approach bridges molecular mechanisms with actionable healthcare strategies, facilitating earlier and more effective interventions. The hypothesis aligns with PPPM principles by emphasizing the potentials of IgG N-glycosylation as novel biomarkers for the early prediction, prevention and intervention of IR.

### Innovation towards the following

#### Predictive approach

This study identified IgG N-glycans, FA2[6]G1 and A2G2S2, as predictive biomarkers for assessing the risk and prevention of IR. The observed associations suggest that these glycans play a crucial role in modulating the immunometabolic axis during IR development. Specifically, FA2[6]G1, previously linked to a lower risk of type 2 diabetes, emerged as a transitional marker in this study, indicating a shift from IR to type 2 diabetes. A2G2S2, characterized by anti-inflammatory properties due to its high sialylation and galactosylation, appeared to mitigate the progression of IR with its stable decline correlating with increasing severity of IR. These patterns suggest the potential of FA2[6]G1 and A2G2S2 as predictive biomarkers that could aid in early detection, precise risk stratification and preemptive interventions [64, 65].

PPPM emphasizes the proactive identification of molecular signatures to predict and prevent chronic disease progression [66, 67]. The significant associations of FA2[6]G1 and A2G2S2 with IR severity, along with their predictive capacity, highlight their promise for future applications in early risk stratification of IR. The observed changes in IgG N-glycosylation, including reduced sialylation and galactosylation, and increased bisecting GlcNAc, were consistent with previous findings in metabolic diseases [65, 68]. This highlights the integral role of altered IgG N-glycans in the pathogenesis and progression of IR, offering insights into the immunometabolic interplay underlying IR. Additionally, these insights align with PPPM principles and open avenues for targeted intervention strategies.

#### Targeted prevention

By characterizing pro-inflammatory glycan traits (e.g., reduced sialylation and galactosylation, increased bisecting GlcNAc) and specific glycans (FA2[6]G1 and A2G2S2) associated with IR, this study supports the development of intervention strategies, including pharmacological strategies or glycoengineering approaches, to modulate IgG N-glycosylation. The interaction between the IgG N-glycans and inflammatory markers, such as TNF-α, IL- 6, CRP, and adiponectin, highlights a complex immunometabolic network underpinning the pathogenesis and progression of IR [8]. Targeting pro-inflammatory glycans of IgG could suppress pro-inflammatory response and attenuate inflammation-driven IR [69]. Notably, the protective roles of glycans FA2[6]G1 and A2G2S2 in IR progression suggest their potential as therapeutic targets. Tailored interventions that address these glycans may not only prevent the worsening of IR but also contribute to broader strategies aimed at reducing the burden of metabolic disorders, thereby advancing the principles of preventive medicine [21, 70]. These findings pave the way for targeted interventions designed to modulate IgG N-glycosylation profiles, reduce inflammation, and lower IR risk.

#### Personalisation of medical services

Precision medicine leverages multidimensional data to optimize disease management, including the prediction, prevention, and treatment of IR. Glycan-targeted therapies, though still in early development, represent a promising approach to mitigating inflammation at the molecular level [70, 71]. Such therapies could complement precise medicine by selectively targeting pro-inflammatory glycans, including those characterized by reduced sialylation and galactosylation or increased bisecting GlcNAc [70, 72]. This study demonstrated that IgG N-glycans influenced IR partially through their modulation of inflammatory markers, suggesting that glycosylation-targeted strategies could provide personalized therapeutic benefits [73]. For personalized strategies, glycoengineering could modify specific glycosylation patterns to reduce pro-inflammatory responses, while anti-inflammatory therapies tailored to glycosylation profiles could address distinct inflammatory and metabolic demands [74, 75]. Additionally, stratifying patients with IR using glycomic and inflammatory marker profiles could enable the development of precise therapeutic and preventive interventions [8, 70]. Tailored therapeutic options targeting IgG N-glycosylation signatures would represent a significant advancement over generalized treatment protocols.

### Contribution to the Paradigm Shift from Reactive Medicine to PPPM

This research exemplifies a paradigm shift from reactive treatments of IR to a proactive healthcare by integrating PPPM principles into IR research. It highlights the potential central role of IgG N-glycosylation in the immunometabolic axis, shedding light on its interaction with inflammatory markers and offering insights into potential mechanistic pathways that may inform future interventions. The identification of both protective and pro-inflammatory glycoforms in this study facilitates stratified risk assessment, enabling more targeted prevention strategies. Moreover, the independent influence of IgG N-glycans on IR, partially through modulation of inflammation, deepens our understanding of how chronic inflammation drives IR [5]. These findings pave the way for innovative interventions, such as glycan-targeted therapies [56], which align with the principles of personalized and preventive medicine [11]. This study bridges fundamental research and clinical application, demonstrating the feasibility PPPM in real-world healthcare settings [76]. By integrating glycomics profiling with inflammatory and metabolic biomarkers, this study underscores the transformative potential of PPPM in addressing complexities of IR.

## Supplementary Information

Below is the link to the electronic supplementary material.

Supplementary file 1 (XLSX 608 KB)

## Data Availability

The datasets generated and/or analysed during the current study are available from the corresponding author upon reasonable request.

## Abbreviations

IR: insulin resistance
PPPM/3PM: predictive, preventive, and personalized medicine
IgG: immunoglobulin G
HOMA-IR: homeostatic model assessment for insulin resistance
LDL: low-density lipoprotein
HDL: high-density lipoprotein
HbA1c: haemoglobin A1c
TNF: tumour necrosis factor
IL: interleukin
CRP: C-reactive protein
Fc: fragment crystallizable
GlcNAc: N-acetylglucosamine
CCA: canonical correlation analysis
FPG: fasting plasma glucose
BMI: body mass index
smCCA: sparse multiset CCA
LASSO: least absolute shrinkage and selection operator
GP: glycan peak
F: α-1, 6-linked core fucose
A_x_: number (x) of antennae
B: bisecting N-acetylglucosamine (GlcNac) β (1–4) linked to β (1–3) mannose
M_x_: number (x) of mannose residues
Gx: number (x) of β(1–4) linked galactoses
[3]G1: galactose on the antenna of the α(1–3) linked mannose
[6]G1: galactose on the antenna of the α(1–6) linked mannose
S_x_: number (x) of sialic acids linked to galactose
OR: odds ratio
ADCC: antibody-dependent cellular cytotoxicity
C1q: complement component 1q
CDC: complement-dependent cytotoxicity
